# Supplementary material for: Sex-dependent effects of chronic intermittent voluntary alcohol consumption on attentional, not motivational, measures during probabilistic learning and reversal
Source: PLoS One. 2020 Jun 18;15(6):e0234729. doi: 10.1371/journal.pone.0234729 (PMC7302450; doi:10.1371/journal.pone.0234729)
Supplement: S1 Fig — (A) All animals regardless of drinking group or sex increased their number of rewards collected over the twenty testing days of discrimination learning. H2O-drinking animals and males collected a greater number of rewards than EtOH-drinking animals and females, respectively. Females displayed a greater increase in rewards collected across days. (B) All animals regardless of drinking group or sex decreased their initiation omissions over the twenty testing days of discrimination learning. EtOH-drinking animals and females had more initiation omissions than H2O-drinking animals and males, respectively. EtOH-drinking females displayed more initiation omissions than H2O-drinking females. (C) All animals regardless of drinking group or sex increased their number of rewards collected over the fifteen testing days of reversal learning. There were no group or sex differences on number of rewards collected. (D) All animals regardless of drinking group or sex decreased the number of initiation omissions over the fifteen testing days of reversal learning. There were no group or sex differences on initiation omissions. Bars indicate ± S. E. M. (DOCX) [file pone.0234729.s001.docx]

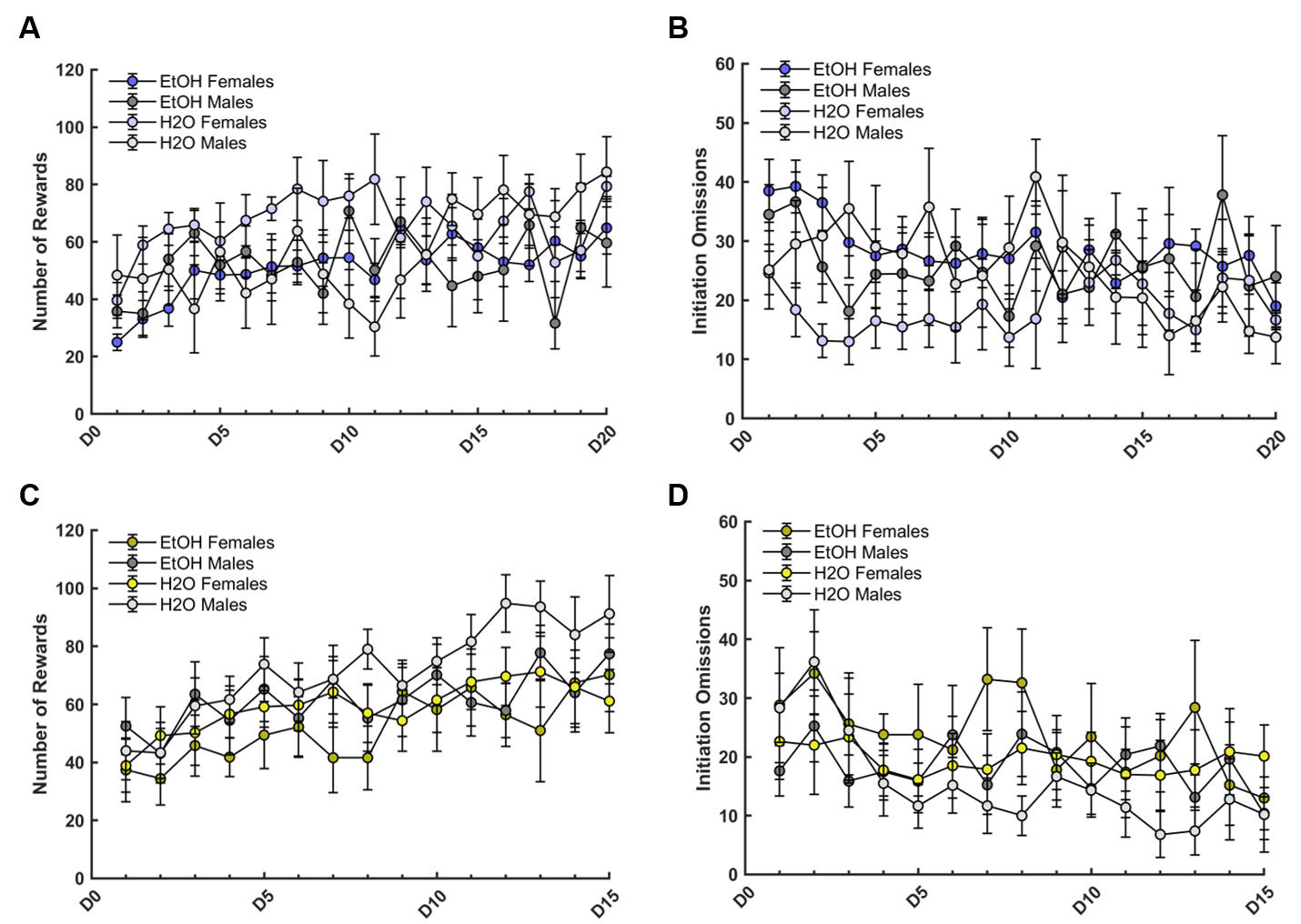


**Fig S1. Drinking group and sex differences on number of rewards and initiation omissions during probabilistic discrimination and reversal learning.** (**A**) All animals regardless of drinking group or sex increased their number of rewards collected over the twenty testing days of discrimination learning. H2O-drinking animals and males collected a greater number of rewards than EtOH-drinking animals and females, respectively. Females displayed a greater increase in rewards collected across days. (**B**) All animals regardless of drinking group or sex decreased their initiation omissions over the twenty testing days of discrimination learning. EtOH-drinking animals and females had more initiation omissions than H2O-drinking animals and males, respectively. EtOH-drinking females displayed more initiation omissions than H2O-drinking females. (**C**) All animals regardless of drinking group or sex increased their number of rewards collected over the fifteen testing days of reversal learning. There were no group or sex differences on number of rewards collected. (**D**) All animals regardless of drinking group or sex decreased the number of initiation omissions over the fifteen testing days of reversal learning. There were no group or sex differences on initiation omissions. Bars indicate $\pm S.E.M.$
