# Supplementary material for: Sex-dependent effects of chronic intermittent voluntary alcohol consumption on attentional, not motivational, measures during probabilistic learning and reversal
Source: PLoS One. 2020 Jun 18;15(6):e0234729. doi: 10.1371/journal.pone.0234729 (PMC7302450; doi:10.1371/journal.pone.0234729)
Supplement: S2 Fig — (A) No group or sex differences in the use of the win-stay strategy. (B) EtOH-drinking animals used the lose-shift strategy more than H2O-drinking animals. Bars indicate ± S. E. M. **p ≤0.01. (DOCX) [file pone.0234729.s002.docx]

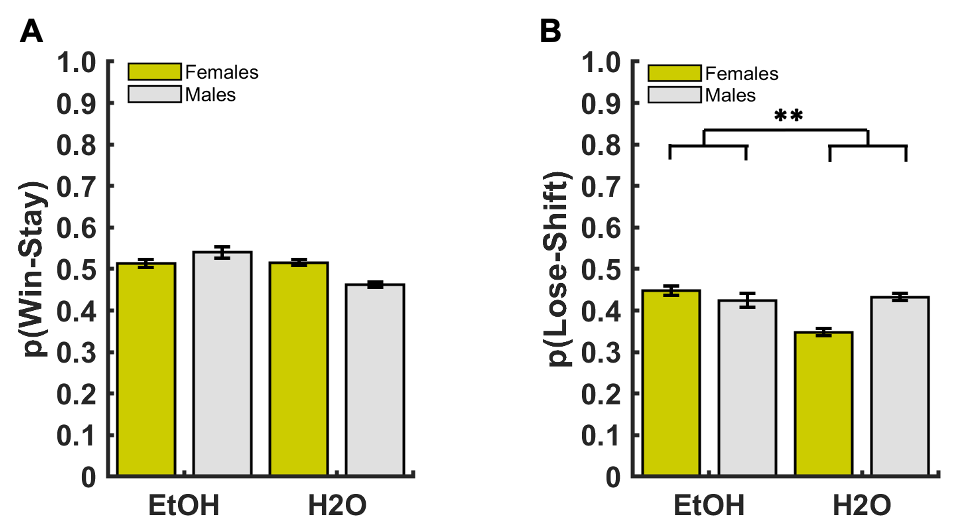


**Fig S2. Drinking group differences in use of lose-shift and lose-stay strategies during early probabilistic reversal learning.** (**A**) No group or sex differences in the use of the win-stay strategy. (**B**) EtOH-drinking animals used the lose-shift strategy more than H2O-drinking animals. Bars indicate $\pm S.E.M$. **p ≤0.01
