# Supplementary material for: Sex-dependent effects of chronic intermittent voluntary alcohol consumption on attentional, not motivational, measures during probabilistic learning and reversal
Source: PLoS One. 2020 Jun 18;15(6):e0234729. doi: 10.1371/journal.pone.0234729 (PMC7302450; doi:10.1371/journal.pone.0234729)
Supplement: S3 Fig — (A) EtOH group exhibited longer initiation latencies than the H2O group. (B) EtOH group exhibited longer forced-choice latencies than the H2O group. (C) No group differences were found for reward latencies. (D) No sex differences were found for initiation latencies. (E) Females exhibited longer forced-choice latencies than males. (F) No sex differences were found for reward latencies. Dashed lines in latency histograms represent group medians. Bars indicate ± S. E. M. n = 16 males, n = 16 females, *p<0.05. (DOCX) [file pone.0234729.s003.docx]

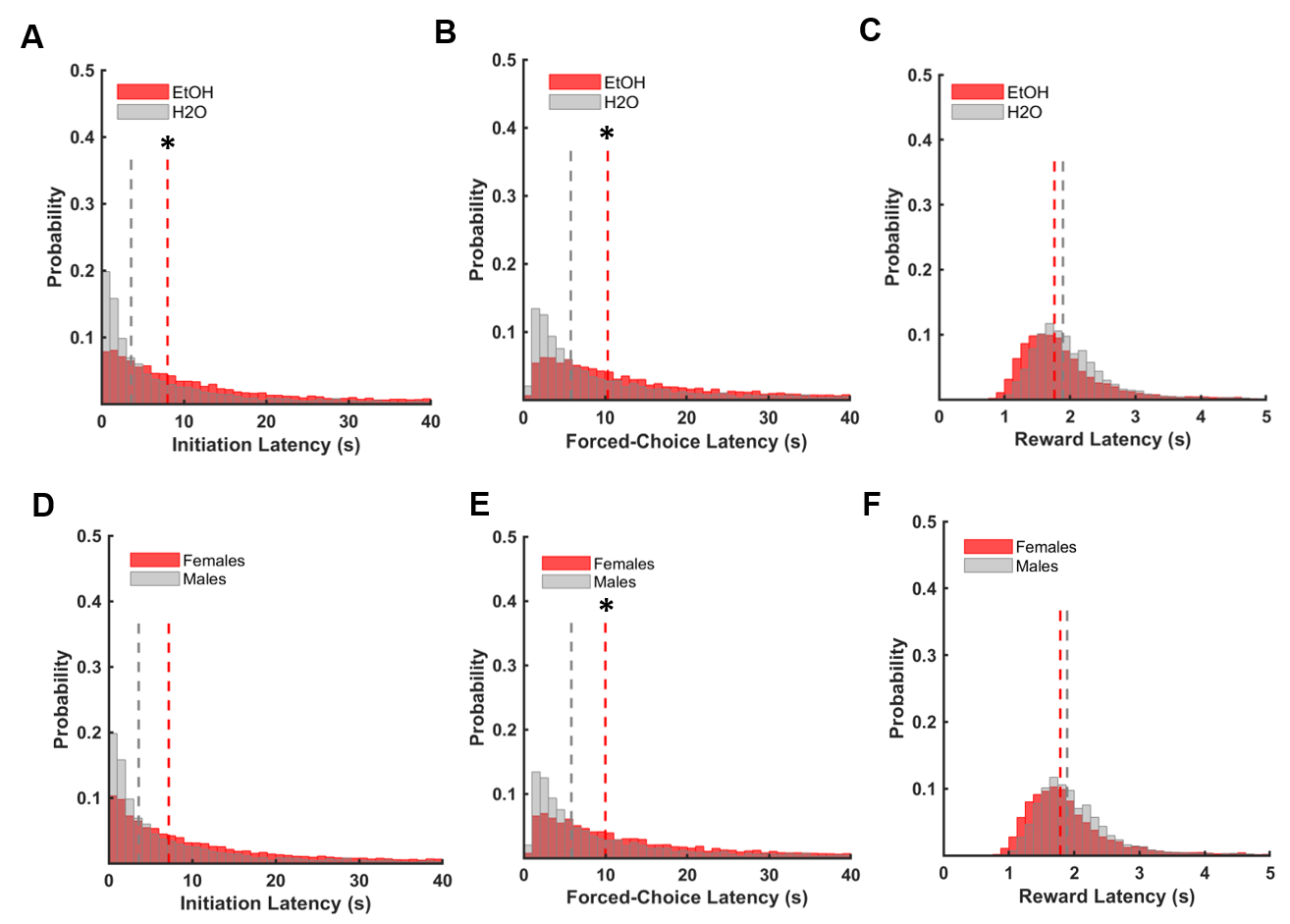


**Fig S3. Drinking group and sex differences in latencies during operant pretraining** (**A**) EtOH group exhibited longer initiation latencies than the H2O group. (**B**) EtOH group exhibited longer forced-choice latencies than the H2O group. (**C**) No group differences were found for reward latencies. (**D**) No sex differences were found for initiation latencies. (**E**) Females exhibited longer forced-choice latencies than males. **(F)** No sex differences were found for reward latencies. Dashed lines in latency histograms represent group medians. Bars indicate $\pm S.E.M.$ n=16 males, n=16 females, *p<0.05
