# Supplementary material for: Sex-dependent effects of chronic intermittent voluntary alcohol consumption on attentional, not motivational, measures during probabilistic learning and reversal
Source: PLoS One. 2020 Jun 18;15(6):e0234729. doi: 10.1371/journal.pone.0234729 (PMC7302450; doi:10.1371/journal.pone.0234729)
Supplement: S5 Fig — (A) No group differences were found for initiation latencies for the first 500 trials. (B) No group differences were found for reward latencies for the first 500 trials. (C) No group differences were found for initiation latencies for the last 500 trials. (D) H2O group exhibited longer reward latencies compared to the EtOH group for the last 500 trials. (E) No sex differences were found for initiation latencies for the first 500 trials. (F) No sex differences were found for reward latencies for the first 500 trials. (G) Females exhibited longer initiation latencies compared to males for the last 500 trials. (H) No sex differences were found for reward latencies for the last 500 trials. Dashed lines in histograms of latencies represent group medians. Bars indicate ± S. E. M. n = 16 males, n = 14 females, *p ≤0.05, **p ≤0.01. (DOCX) [file pone.0234729.s005.docx]

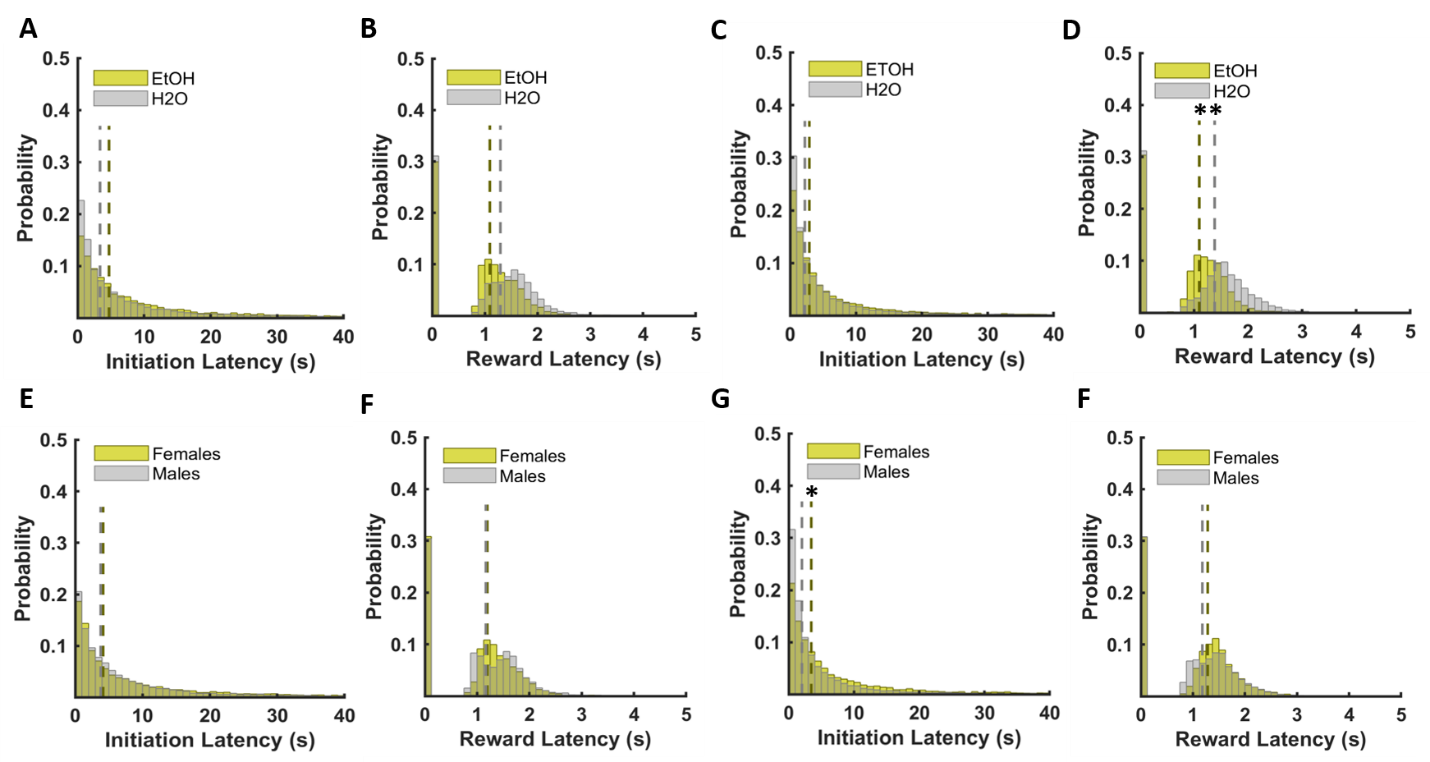


**Fig S5. Drinking group and sex differences in latencies during early and late probabilistic reversal learning.** (**A**) No group differences were found for initiation latencies for the first 500 trials. (**B**) No group differences were found for reward latencies for the first 500 trials. (**C**) No group differences were found for initiation latencies for the last 500 trials. (**D**) H2O group exhibited longer reward latencies compared to the EtOH group for the last 500 trials. (**E**) No sex differences were found for initiation latencies for the first 500 trials. (**F**) No sex differences were found for reward latencies for the first 500 trials. (**G**) Females exhibited longer initiation latencies compared to males for the last 500 trials. (**H**) No sex differences were found for reward latencies for the last 500 trials. Dashed lines in histograms of latencies represent group medians. Bars indicate $\pm S.E.M.$ n=16 males, n=14 females, *p ≤0.05, **p ≤0.01
